# SARS-CoV-2 in Myelodysplastic Syndromes: A Snapshot From Early Italian Experience

**DOI:** 10.1097/HS9.0000000000000483

**Published:** 2020-09-23

**Authors:** Sandra Mossuto, Enrico Attardi, Francesco Alesiani, Emanuele Angelucci, Enrico Balleari, Massimo Bernardi, Gianni Binotto, Costanza Bosi, Anna Calvisi, Isabella Capodanno, Antonella Carbone, Andrea Castelli, Marco Cerrano, Rosanna Ciancia, Daniela Cilloni, Marino Clavio, Cristina Clissa, Elena Crisà, Monica Crugnola, Matteo G. Della Porta, Nicola Di Renzo, Ambra Di Veroli, Roberto Fattizzo, Carmen Fava, Susanna Fenu, Ida L. Ferrara, Luana Fianchi, Carla Filì, Carlo Finelli, Valentina Giai, Francesco Frattini, Valentina Gaidano, Gianluca Guaragna, Svitlana Gumenyuk, Roberto Latagliata, Stefano Mancini, Emanuela Messa, Alfredo Molteni, Pellegrino Musto, Pasquale Niscola, Esther Oliva, Giuseppe A. Palumbo, Annamaria Pelizzari, Federica Pilo, Antonella Poloni, Marta Riva, Flavia Rivellini, Chiara Sarlo, Mariarita Sciumé, Roberto Secchi, Carmine Selleri, Agostino Tafuri, Valeria Santini

**Affiliations:** 1 Italian MDS Foundation - ETS (FISIM - ETS), Bologna, Italy; 2 Hematology, University of Florence, AOU Careggi, Florence, Italy; 3 Hematology and Transplant Center, Area Vasta 3 Macerata-ASUR Marche, Ospedale di Civitanova Marche, Italy; 4 Hematology and Transplant Center, IRCCS Policlino San Martino Hospital, Genova, Italy; 5 Internal Medicine- Azienda Sanitaria locale 1 Imperiese- Imperia, Italy; 6 Hematology and Bone Marrow Transplantation, IRCCS San Raffaele Scientific Institute, Milan, Italy; 7 Unit of Hematology and Clinical Immunology, University of Padova, Padova, Italy; 8 Division of Hematology, AUSL di Piacenza, Piacenza, Italy; 9 Hematology Division and Bone Marrow Transplantation Unit, San Francesco Hospital, Nuoro, Italy; 10 Hematology Unit, Azienda Unità Sanitaria Locale-IRCCS, Reggio Emilia, Italy; 11 GROM-L (Gruppo Romano-Laziale MDS), Italy; 12 Hematology Unit, Presidio Ospedaliero di Frosinone, Italy; 13 Division of Hematology, Ospedale Degli Infermi, Biella, Italy; 14 Division of Hematology, University of Torino, AOU Città della Salute e Della Scienza, Torino, Italy; 15 Unit of Onco-hematology, Hematopoietic Transplants and Cell Therapies, Centro di Riferimento Oncologico di Aviano (CRO) IRCCS, Italy; 16 Department of Clinical and Biological Sciences of the University of Turin, San Luigi Hospital, Orbassano, Turin, Italy; 17 Clinic of Hematology, Department of Internal Medicine (DiMI), University of Genoa, Genoa, Italy; 18 Hematology and Hematopoietic Stem Cell Transplant Center, AORMN (Azienda Ospedaliera Ospedali Riuniti Marche Nord), Pesaro, Italy; 19 Division of Hematology, Department of Translational Medicine, Università del Piemonte Orientale and Ospedale Maggiore della Carità, Novara, Italy; 20 Hematology Unit and BMT Center, Azienda Ospedaliero Universitaria di Parma, Parma, Italy; 21 Cancer Center, Humanitas Research Hospital and Humanitas University, Milan, Italy; 22 Hematology and BMT Unit, Ospedale Vito Fazzi, Lecce, Italy; 23 Hematology Unit Ospedale. Bel Colle-Viterbo, Italy; 24 Hematology Unit, Fondazione IRCCS Ca’ Granda Ospedale Maggiore Policlinico, Milan, Italy; 25 Department of Clinical and Biological Sciences of the University of Turin, Mauriziano Hospital, Italy; 26 Hematology Department, AO, San Giovanni-Addolorata, Rome, Italy; 27 Department of Medicine and Surgery, University of Salerno, Salerno, Italy; 28 Hematology Unit, Università Cattolica del Sacro Cuore (UCSC) Roma, Italy; 29 Clinical Hematology, Transplant Center and Cell Therapy, Azienda Sanitaria Universitaria Integrata di Udine, S. Maria della Misericordia, Udine, Italy; 30 UO Hematology, AOU Policlinico Sant’Orsola-Malpighi, University of Bologna, Bologna, Italy; 31 Division of Hematology, Department of Oncology, AOU Città della Salute e della Scienza, Turin, Italy; 32 Department of Hematology and Transfusion Medicine, Carlo Poma Hospital, Mantova, Italy; 33 Hematology, SS.Antonio, Biagio e Cesare Arrigo Hospital, Alessandria, Italy; 34 Hematology and BMT Unit-“Antonio Perrino” Hospital, 72100 Brindisi, Italy; 35 Hematology and Stem Cell Transplantation Unit, Regina Elena National Cancer Institute IRCCS-IFO - Rome, Italy; 36 Hematology Department, University La Sapienza, Rome, Italy; 37 Hematology Unit, AO San Camillo-Forlanini, Rome, Italy.; 38 UO Internal Medicine, ASLTo4, Carmagnola, Italy; 39 Hematology Unit, ASST Cremona, Cremona, Italy; 40 Chair of Hematology and Unit of Hematology and Stem Cell Transplantation, “Aldo Moro” University School of Medicine, AOU Consorziale Policlinico, Bari, Italy.; 41 Hematology Unit, Sant’Eugenio Hospital, Rome, Italy; 42 UO Hematology, Grande Ospedale Metropolitano, “Bianchi Melacrino Morelli”, Reggio Calabria, Italy; 43 Department of Scienze Mediche Chirurgiche e Tecnologie Avanzate, “G.F. Ingrassia”, University of Catania, Italy; 44 Hematology, ASST-Spedali Civili, Brescia, Italy; 45 Hematology and Transplant Center, Ospedale Oncologico “Armando Businco” Cagliari, Italy; 46 Hematology, Polytechnic University of Marche, AUO Ospedali Riuniti, Ancona, Italy; 47 Hematology, ASST Grande Ospedale Metropolitano Niguarda, Milan, Italy; 48 Onco-Hematology, “A. Tortora” Hospital, Pagani (Sa), Italy; 49 Hematology and Stem Cell Transplantation Unit, University Campus Bio-Medico, Rome, Italy; 50 Hematology Division, University of Tor Vergata, Rome, Italy.; 51 Hematology Institute, La Sapienza University of Rome, S. Andrea Hospital, Rome, Italy.

Myelodysplastic syndrome patients are subjects of advanced age, vulnerable and frail, whose outcome is heavily influenced by pre-existing comorbidities worsening the hematologic condition. Infections are a rather common cause of death (around 30%), especially, but not only, for IPSS-R higher risk patients.^1–3^ In MDS there is a significant impairment of lymphopoiesis, resulting in lymphopenia (ALC < 1.0 × 10^9^/l) in around 38% of MDS patients and poor prognosis.^4^ Data on innate and adoptive immune systems (either disease related or due to immunosenescence) and the subsequent supposed susceptibility and incidence of viral infections in MDS are scarce.^5^

With all these considerations in mind, at Coronavirus outbreak in Italy, the spread of the COVID-19 pandemic was so extremely rapid that we were expecting to face in short times a very large number of severely symptomatic MDS patients and tried to rapidly learn from the earlier and more severely hit areas.^6^ As per April 28, date until which we collected our data, and in full emergency, the number of diagnosed cases of SARS-Cov2 in Italy was 199.470 with 25.215 deaths.^7^ In the same period, in the general population around 10% of tested cases were actually infected.^8^

Collection of data in the national MDS Registry (FISiM) and the regional Registry for Rome and surrounding area (GROM) has been approved by local Ethical Committees. Through these 2 networks we collected data regarding laboratory-confirmed severe acute respiratory syndrome coronavirus 2 (SARS-CoV-2) in MDS patients symptomatic and tested from February 24 to April 28, 2020. Data have been obtained from 50 Centers. Total number of MDS patients followed up in that period was 5326 as per April 28, median age 73 years. As per national guidelines, oropharingeal and nasal swab with PCR was performed in regional laboratories for all suspected cases: 305/5326, tested irrespective of gravity of symptoms, and the presence of true SARS-CoV-2 infection sent for confirmation in the national reference central laboratory (Istituto superiore di sanità -ISS). Confirmed SARS-CoV-2 was diagnosed in 63/305 tested cases (20.6%), globally in 63/5326 (1.18%) MDS patients, in the time frame indicated above. Median age of affected MDS patients was 78 years. We evaluated the distribution of SARS-CoV-2 cases dividing the Country into three macro-regions, considering adhering Centers and the epidemiology and cumulative incidence of COVID-19 in Italy:^7^ 3 Regions of Northern Italy (Lombardia, Piemonte and Emilia Romagna, SARS-CoV-2 > 500 cases /100.000 inhabitants, as per April 28), Rome and surroundings (specific GROM Registry), and Rest of Italy (Table 1). The majority of SARS-CoV-2 cases and cumulative incidence among MDS patients was localized in the 3 Regions of Northern Italy (LPE) (1.6%), consistent with the data of COVID-19 epidemic in the general population of the area (62.4% of total Italian cases),^7^ while in Rome and Rest of Italy it was < 1%, (0.3 and 0.85% respectively). Median age of affected MDS patients in LPE Regions was 81 years, in Rome 71 years and in the rest of Italy 77 years.

**Table 1:**
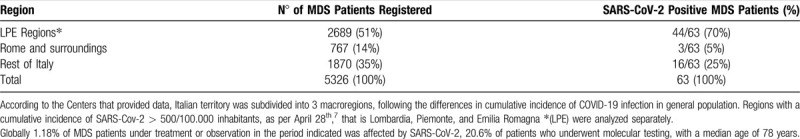
Distribution of SARS-CoV-2 Positive MDS Patients Diagnosed from February 24th to April 28th 2020, Respect to Number of MDS Patients in Treatment in 50 Italian Hematology Centers.

At the time of analysis, only 33/63 patients were alive, indicating a lethality rate significantly higher than that of non–MDS population (same age range 70–79 years: 28.9% deceased, lethality 24%).^7^

Available details on demographics, clinical characteristics and treatment of 63 MDS patients with SARS-CoV-2 are indicated in Table 2. It is evident that SARS-CoV-2 affected prevalently male subjects, confirming the observation in non-MDS Italian affected population aged 70 to 79 yrs. In particular, although numbers are extremely small, the lethality rate was higher in male MDS patients (73% of total deaths). To note, the same trend was noted for male patient in the general population infected (lethality for male 29.5% vs 16.7% for female aged 70–79 years),^7^ while survival of MDS patients was not apparently influenced by age (median age 78 years in both groups). Reported cause of death for all 30 cases was respiratory failure, in 82% of cases COVID-19 was complicated by bacterial pneumonia and 5% cardiac failure. ARDS was indicated in 50% of deceased cases. Regarding IPSS-R risk categories, the majority of patients who recovered were lower risk ones (62%), while deceased patients were in the great majority IPSS-R higher risk ones (17/30). There is no statistically significant difference for infection, gravity of infection or survival according to the type of treatment received, in part due to the small figures when we come to the granularity of therapies. A higher proportion of patients was in treatment with azacitidine, consistent with their diagnosis of IPSS-R higher risk MDS. The totality of the MDS patients who were diagnosed with SARS-CoV-2 had multiple severe comorbidities (> 3 comorbidities 80% of cases).

**Table 2:**
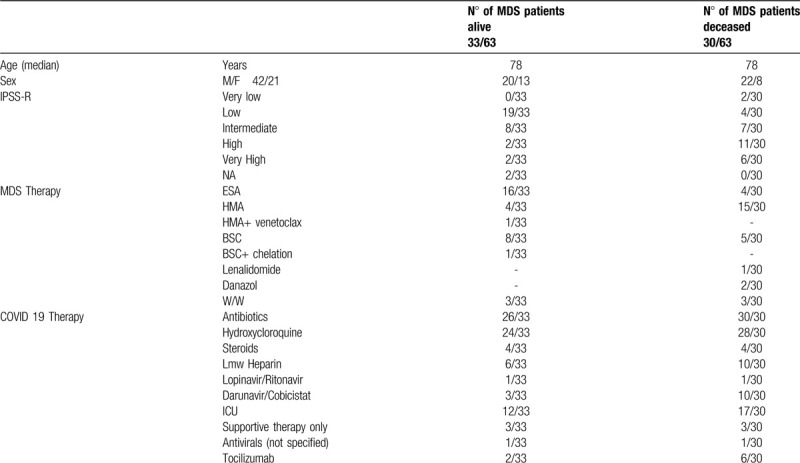
Demographics and Clinical Characteristics of MDS Patients Diagnosed with SARS-CoV-2 and Available Data on MDS and COVID-19 Treatment.

A few patients received only supportive care for COVID-19 infection, either for milder clinical presentation (3/33) or, on the contrary, for a rapid and extremely aggressive onset leading to early death (3/30). In the majority of cases, MDS specific therapies were suspended.

The impact of SARS-CoV-2 on the frail MDS population was evaluated in a limited time frame during the peak of the pandemic in Italy and the strict national lockdown. Incidence of symptomatic infection was not as relevant as expected in MDS patients for whom neutropenia, lymphopenia, stress erythropoiesis and iron overload could have determined a substantial susceptibility to and gravity of SARS-CoV-2. Similar observations were recently reported, in a much younger population of beta thalassemic patients.^9^ Median age of SARS-CoV-2 MDS patients was higher than that of the affected Italian MDS population, and this, together with comorbidities, may account for the high lethality rate observed.^10^ This report is limited and preliminary (early landmark date), produced during the health emergency. Here we share the international problem of general epidemiology of SARS-CoV-2. In fact, we do not have data for asymptomatic infected MDS patients, for whom diagnostic procedures were not performed, and still complete data are lacking. At present, after resolution of the health emergency, routine serology evaluation of COVID-19 antibodies is ongoing for MDS patients managed in our Centers.
